# Connecting the person with dementia and family: a feasibility study of a telepresence robot

**DOI:** 10.1186/1471-2318-14-7

**Published:** 2014-01-24

**Authors:** Wendy Moyle, Cindy Jones, Marie Cooke, Siobhan O’Dwyer, Billy Sung, Suzie Drummond

**Affiliations:** 1 Griffith Health Institute, Griffith University, 170 Kessels Road, Brisbane QLD 4111, Australia; 2 Centre for Health Practice Innovation, Griffith University, 170 Kessels Road, Brisbane QLD 4111, Australia; 3 School of Nursing and Midwifery, Griffith University, Nathan Campus, 170 Kessels Road, Brisbane QLD 4111, Australia; 4 Dementia Collaborative Research Centre – Carers and Consumers, QUT, Brisbane, Australia

**Keywords:** Dementia, Telepresence robots, Communication, Family, Long-term care, Technology

## Abstract

**Background:**

Maintenance of communication is important for people with dementia living in long-term care. The purpose of this study was to assess the feasibility of using “Giraff”, a telepresence robot to enhance engagement between family and a person with dementia living in long-term care.

**Methods:**

A mixed-methods approach involving semi-structured interviews, call records and video observational data was used. Five people with dementia and their family member participated in a discussion via the Giraff robot for a minimum of six times over a six-week period. A feasibility framework was used to assess feasibility and included video analysis of emotional response and engagement.

**Results:**

Twenty-six calls with an average duration of 23 mins took place. Residents showed a general state of positive emotions across the calls with a high level of engagement and a minimal level of negative emotions. Participants enjoyed the experience and families reported that the Giraff robot offered the opportunity to reduce social isolation. A number of software and hardware challenges were encountered.

**Conclusions:**

Participants perceived this novel approach to engage families and people with dementia as a feasible option. Participants were observed and also reported to enjoy the experience. The technical challenges identified have been improved in a newer version of the robot. Future research should include a feasibility trial of longer duration, with a larger sample and a cost analysis.

## Background

One of the most important aspects of dementia care is the maintenance of communication between people with dementia, family and staff so that care provision can be appropriately individualised [1]. The loss of in-depth communication, as well as social conversations, can result in the person with dementia feeling socially isolated and without the opportunity to express their needs [2]. Communication is further challenged by the disease state; people in the advanced stages of dementia lose the ability to express emotions verbally. Recent research however, has shown that analyses of non-verbal behaviours are an important means to assess emotional states in people with dementia and can help staff and family to individualise attention in order to encourage positive emotional states [3].

When the person enters long-term care, communication opportunities with family can also be further reduced. This is particularly the case in situations: where work pressures challenge family; the distance to the long-term care facility may inhibit opportunity to spend time with their family member; and, family may feel they have a limited role in the provision of care [4,5]. Opportunities to connect families and people with dementia living in long-term care may now be made possible through new technologies such as telepresence robots. The aim of this study was to assess the feasibility of using such technology to enhance engagement between the person with dementia living in long-term care and their family.

### Telepresence robots

Telepresence robots were first described in the literature as a human-machine interface. Telepresence robots do not possess autonomous behaviours and therefore they require a remote user to operate them. Through the telepresence technology, the operator can virtually see into another space through a two-way camera and can operate the robot via software on their computer. The operator can therefore feel they are physically present at the location of the robot no matter where the robot is located [6]. Recent advances in information and communication technologies have facilitated the development of telepresence robots for consultations in healthcare and teaching settings [7,8]. However, given the newness of this technology, there are few noteworthy research papers available and discussions on the use of such technologies with older people have only recently surfaced.

The concept of a telepresence robot for in-home care for older people first appeared in the literature in 2007. Independently a group from Canada examined the requirements for an in-home telepresence robot through focus group discussion with six older people and six healthcare professionals. This qualitative research identified potential applications of opportunities where older people might use such a robot, such as to connect with staff and family [9]. A team from Taiwan developed a prototype telepresence robot named “TRIC” (Telepresence Robot for Interpersonal Communication) to allow older people to remain at home while family and caregivers communicate with and monitor their older family members’ safety and health. “TRIC” was tested in a laboratory environment [10]. According to the researchers, “TRIC” enabled an older person to recognise the telepresence robot as a representation of the operator, for example family, and this is thought to lead to effective communication.

Further developments have been advanced in the area of health consultation. The Physician-Robot is a 5-foot-tall telepresence robot equipped with a real-time video display on its flat-screen head. This robot is designed to facilitate easier and more frequent interactions between physicians and their hospitalised patients. Ellison and colleagues [11] tested the effectiveness of the Physician-Robot with patients who required postoperative care. Patients, who were offered robotic tele-visit bedside rounds, reported substantial improvements in quality of care when compared with patients who received a standard once daily bedside round with the physician. Enhancements were observed in ratings of examination thoroughness, quality of discussions about medical information, postoperative care coordination, and satisfaction with physician availability. Similar results were also reported in another study, where 80 per cent of study participants indicated marked improvements in physician availability and interaction quality.

A telepresence robot designed specifically for older and disabled people includes the Giraff (Giraff Technologies AB) [12] and VGo (VGo Communications Inc.) [13]. VGo was originally designed for communication in business but VGO is now being used in hospital and assisted living communities. Both Giraff and VGo are also being tested in long-term care by the authors of this paper. Long-term care refers to facilities such as nursing homes that provide healthcare to people who are unable to manage in the community.

The most recent and advanced feasibility investigation of telepresence robots is taking place in a European project called ExCITE (Enabling Social Interaction Through Embodiment) [14]. The Giraff robot is being tested by the ExCITE team [15]. Giraff is a remotely controlled, mobile, human-height, telepresence robot. The researchers aim to develop and refine a prototype of Giraff through the involvement of end users and to explore the use of it for addressing social isolation and loneliness. The program also seeks to examine the interaction between the users (e.g. users, family, physician, and relevant organisation) and the telepresence system [15].

Giraff is currently manufactured in Europe and is equipped with a videoconferencing system that includes a video camera, LCD screen, speaker and microphone. Giraff is battery powered with a charge lasting approximately one hour. A docking station charges the battery in around two hours. A standard computer with Giraff software allows the user to move Giraff by holding down the left button on a standard computer mouse while pointing to a place within the environment. This action will enable Giraff to move to the place indicated on the user’s computer screen. Giraff is intended to move forward but can turn in a circle as well as move backwards if it becomes stuck, for example, on a rug. The base of Giraff moves using a differential drive system. Giraff weighs 14 kg, enabling it to be easily transported as well as carried up stairs using an inbuilt carrying handle. It cannot however, climb stairs or inclines. The large video screen allows the user’s face to be near life size and the head can be tilted and moved sideways to simulate eye contact, as well as to control the field of view. During the testing of Giraff end users reported concerns in relation to privacy, which resulted in the development of a database to manage who has access to Giraff and a call button so that the user can respond or cancel calls. Giraff cannot directly connect to a cellular network to provide Internet access and relies on a USB wireless adapter (dongle) that is connected to one of Giraff’s exterior USB ports. This allows Giraff to connect to any available wireless network that provides Internet access and to establish calls. For the current project we added an external modem to Giraff to receive the cellular network from the telecommunication provider and the modem’s inbuilt router created and transmitted a secured wireless network for Giraff to connect to the Internet.

In the ExCITE project, further research proposed by European researchers Tiberio et al. [16] plans to compare the implication of Giraff between a treatment group of five older people with Mild Cognitive Impairment and a control group of five hospitalised older people without MCI. Over three phases, the researchers will introduce participants to Giraff, demonstrate its functions, and facilitate basic communications between participants and researchers. Participant perceptions of social engagement, perceived utility and privacy will be assessed in an interview at the end of the project [16]. Physiological markers of stress and measures of anxiety will also be collected. While Tiberio and colleague’s [16] research will provide an important insight into the use of telepresence robots for older people, the study lacks practical applicability as communication will be with a researcher situated in another room rather than a genuinely remote family member or loved one.

The current project described in this paper used the Giraff telepresence robot to connect a family member and a person with dementia as a means of enhancing communication between these two parties. Using this videoconferencing, ‘skype-onwheels’ like system, families can ‘virtually’ visit people with dementia – engaging in two-way conversations, with their face appearing on Giraff’s ‘life size’ video screen for the person with dementia and allowing the family to view the person and their surroundings. Using such technology the family member can, from anywhere in the world, use their computer to virtually drive Giraff to the resident as well as anywhere within the long-term facility that offers a flat surface. There are many potential situations in which telepresence robots could be used to support older people and promote social interaction. However, the newness of this technology means there are limited studies that have tested the feasibility and the effectiveness of this particular technology in an older population and in particular with people with dementia living in a nursing home environment. This current project will add to information gathered in the ExCITE project as this project was conducted outside of Europe.

This study aimed to explore the use of a Giraff telepresence robot as a means of positively influencing communication and relationships between residents with dementia living in a long-term care facility and their family, and to examine the feasibility (according to the Bowen Feasibility Framework [17] as outlined p. 9) of implementing a Giraff robot in a long-term care facility with older people with cognitive impairment. A feasibility study is an analysis of the viability of an idea, and in this case whether to use Giraff in a long-term care facility. As there is limited research in the use of telepresence robots it is important that small-scale projects such as this are made available so that researchers and clinicians are able to consider the use and further trial of this type of technology as well as the potential factors involved in the trial of telepresence robots in such settings.

## Methods

### Design

A mixed-methods approach involving semi-structured interviews and observational data was used to assess the feasibility of using Giraff to connect the person with dementia with their family member. Analyses of both verbal and non-verbal behaviour aimed to assess emotional states and engagement of the person with dementia. Ethics approval to conduct the trial was received from Griffith University’s Human Research Ethics Committee (NRS/39/12/HREC) and the long-term care facility formally endorsed the research. All participants were provided with written informed consent materials and the opportunity to discuss the research, and to ask questions. Consent or assent for participation was received from both the person with dementia and their family or guardian.

### Participants

Participants were recruited by a representative from one long-term care facility owned and operated by a large not-for-profit provider in Queensland, Australia, that has close research connections with the authors and is situated in an area covered by adequate internet reception and Internet access speed. The care manager introduced the researchers to potential participants and they discussed the research requirements, obtained consent, and arranged the practice and trial calls. The participants were five dyads. Each dyad consisted of a resident from a long-term care facility with mild to moderate stage dementia who were considered by staff to be capable of verbal conversation and comprehension (i.e. they had no significant hearing loss and in spite of cognitive impairment they were thought to be capable of engagement), and a family member (six family members in total as two family members wanted to be involved for one of the residents). In addition seven staff members at the long-term care facility who had been involved or observed the telepresence interaction were asked to participate.

### Intervention

Families were provided with the Giraff dedicated communication software, a procedure manual and training in how to download the communication software onto their computer, connect to Giraff and ‘drive’ Giraff. Family members underwent a practice call with a research team member (BS) to ensure they were able to connect to Giraff from their computers, adjust the field settings, and move Giraff around. The Giraff was then relocated to the long-term care facility for the duration of the study. All calls were made from the family member’s home and received in the long-term care facility, either in the residents’ rooms, a quiet common area, or a quiet closed-off room, depending on the quality of the Internet reception available. All calls were made over a four-month period in 2012–2013. Each resident-family dyad participated in the trial over a six- to eight-week period with the aim of conducting six calls per dyad. Due to technical difficulties, timing issues, and inclement weather (e.g., flash flooding), it was not possible for some resident-family dyads to conduct all six calls, however, all successful calls they made were included in the analysis. Families were advised to conduct the call for between 15–60 minutes, with actual timing dependent on the individual situations. To assist with analysis the duration of each call was standardised into five segments (each reflecting a given 20 per cent of the call) from the start of an established call to the end.

It was initially intended that the long-term care facility staff would be responsible for setting up Giraff and troubleshooting potential problems. However, at the beginning of the trial, it became apparent that troubleshooting the technical difficulties required a greater in-depth knowledge of Giraff than facility staff possessed. Additionally, some of the difficulties took considerable time to address and it was not feasible for staff to spend time addressing these problems in addition to their normal duties. For these reasons, the research team took responsibility for setting up the Giraff and dealing with any technical difficulties.

### Data collection

Referring to Table 1, three sources of data were utilised to provide triangulated information to add strength and credibility to the findings [18]. Table 1, adapted from the Bowen Feasibility Framework, describes the five key areas of focus that the researchers took to address the research questions and to assess the outcomes of interest [17]. The research focused on six areas in the Bowen Framework: acceptability, implementation, practicality, integration, efficacy and adaptation. As the research was a short pilot project it did not consider demand for Giraff, nor did the team consider expansion as this area aims to consider an already successful intervention (see Table 1).

**Table 1:** Key areas of focus, outcomes of interest and data sources

**Area of focus**	**Description**	**Study questions**	**Outcomes of interest**	**Data sources**
Acceptability & Integration	How the participants and the staff and family react to using Giraff	To what extent is the Giraff suitable to implement in a long-term care facility?	Perceived acceptability	Interviews with Family (n = 6) & staff (n = 7)
Implementation & Practicality	The likelihood the Giraff can be implemented as planned and delivered when resources, time and commitment are constrained	To what extent can the Giraff be successfully implemented with participants?	Degree of errors, resourcing, factors influencing implementation (e.g. staff time)	Trial data log and Researcher log
Efficacy	The reactions of participants to using Giraff	To what extent does Giraff show promise of encouraging engagement and positive mood change in people with dementia?	Evidence of trends in predicted direction of mood change	Video observations
Adaptation	Is there a need to change or adapt Giraff for the environment?	To what extent can Giraff be used in its current state?	Degree of errors	Trial data log and research team reflections

### Video recordings

Video recordings of the residents were recorded through the Giraff camera. Due to problems with the call connection and recording software, one resident-family dyad did not have any useable video recordings. All other dyads videos were used in the analysis.

### Semi-structured interviews

The overall aim of the staff and family member interviews was to gauge their perceptions of the feasibility of Giraff within a long-term care facility. Interviews were semi-structured and guided by an interview prompt sheet. Interview questions included “What were your perceptions of Giraff pre and post the research?”; “What helped or didn’t help you to communicate through Giraff?”; “Did you experience any challenges when using Giraff?”; “What do you perceive are the advantages and disadvantages of using Giraff in long-term care?”; “What impact did Giraff have on resident/family member?”. Interviews were conducted at the facility in a private room or on the phone (for interstate and overseas family), and they ranged from 15 to 30 minutes in length. All interviews were digitally recorded and transcribed prior to analysis.

### Research team observations and notes

The type and frequency of technical difficulties encountered were recorded, as well as the steps taken to address them. Any other issues or observations that may have impacted on the trial and therefore have bearing on the experiences of the resident-family dyads or staff members were also recorded.

### Data analysis

The semi-structured interviews were analysed using a thematic analytic approach to reveal themes or issues of importance [19]. Analysis of the data collected through interviews involved reading the full interview transcript; performing a line-by-line analysis and comparison with and between transcripts; identification of similar and dissimilar themes; clustering of themes; re-reading the full transcripts and checking the credibility of themes by two members of the research team [19]. Video recordings were analysed by two independent coders (BS, CJ) using the Noldus ObserverXT 11.5 program [20], which allows users to code observational data in millisecond intervals.

Analyses were only conducted on video recordings of successful call connections. A coding protocol (discussed below) was developed to facilitate the identification of facial emotional responses, verbal engagement, visual alertness, and the use of visual cues (e.g. photographs and the long-term care environment) during communication. The two coders viewed the recordings of each resident’s first call session to gain a comprehensive overview of the resident’s typical verbal and non-verbal behaviours and emotional expressions. The overview served as a calibration for the coding to enhance reliability and validity of the analyses. Inter-rater reliability of the video analyses was exceptionally high (94%) when comparing both the frequency and the sequence of behaviours coded within a one-second-tolerance interval. Furthermore, the optimal intra-rater reliability of 95 per cent was also obtained across all of the dependent measures. The researchers were mindful that visual expression is just one part of the human picture and therefore the coders’ observations were interpreted in the context of the interviews and coding data log.

### Facial emotional responses

The protocol for the coding of facial emotional responses was based on the ‘Observed Emotion Rating Scale’ (OERS; [21]). The OERS was developed to assess affective states (positive and negative emotion) in older people with Alzheimer’s disease. As per the OERS, residents’ emotional responses were categorised as pleasure, anger, anxiety or fear, and sadness, and coded according to frequency. The occurrence of an emotion was coded only if a resident exhibited a unique emotional expression within a given five second timeframe. If the same emotion re-occurred in the same five-second timeframe, it was disregarded and only one instance was coded. However, two or more occurrences were coded if the same emotion persisted for longer than five seconds. Each emotion could therefore be coded to a maximum of twelve occurrences in every minute of the conversation. As previously indicated, to standardise the duration of the calls, each call was proportioned into five segments, where each segment represent a given 20% of the call’s duration. The occurrence of emotional responses in each segment was then averaged across all residents’ calls.

### Engagement

The duration of residents’ engagement was measured by their visual alertness and verbal engagement. Alertness and attention are indicators of non-verbal engagement for people with dementia [22]. Visual alertness was operationalised as the duration for which the resident visually appeared to be alert. Residents were coded as being alert when they appeared to the video analysts to be interested in the conversation, for example they were viewed as watching and interacting with the Giraff screen, and maintaining eye contact with the family member on Giraff. Residents were coded as being *not* alert when they appeared to be disinterested, such as not watching the Giraff screen and avoiding eye contact with the family member. Eye contacts were manually coded and the high inter-rater reliability shows that the coding was consistent. In addition, the duration of residents’ verbal engagement was also measured. Residents were coded as being verbally engaged when they were participating in and maintaining conversation by verbally responding to, or initiating, statements or questions [23]. Conversely, residents were coded as being verbally unengaged when they were not responding or participating in the conversation. Overall, a resident was classified as *engaged* if they appeared to be both visually alert and verbally engaged. To provide a conservative analysis, the occurrence of only one of the two behaviours was coded as *unengaged* as in the absence of visual alertness and verbal engagement the person can be considered to be disinterested in the conversation.

### Visual cues

Giraff allows moveable telepresence interaction and as such participants can show each other items of interest such as objects in their room, the facility garden, or introduce them to staff. To help our understanding of the benefits of Giraff we coded each unique instance where the resident or their family used the video screen to encourage, interact in, and maintain a conversation by incorporating visual stimuli.

## Results

A total of five residents, six family and seven staff participants served as the main identifiers of feasibility. The family and staff participated in semi-structured interviews and the five residents participated in video recordings of their conversations. The demographic characteristics of these groups are displayed in Table 2. The majority of residents were female and in the early to mid-stage of dementia. Family members were predominately daughters and 50 per cent of them lived more than five hours driving time from the facility.

**Table 2:** Demographics and characteristics of participants

			**ID code**	**Sex**	**Age (Years)**	**Time in facility (Years)**	**Staff position**
Dyads							
	Dyad 1						
		Resident	R1	F	84	6	
		Family	F1	F	55		
	Dyad 2						
		Resident	R2	F	79	1.5	
		Family	F2	F	56		
	Dyad 3						
		Resident	R3	M	89	1	
		Family	F3	F	43		
	Dyad 4						
		Resident	R4	F	84	2	
		Family	F4	M	53		
	Dyad 5						
		Resident	R5	F	89	3	
		Family	F5(a)	F	57		
		Family	F5(b)	M	62		
Staff							
		Staff 1	S1	F	54		Lifestyle manager
		Staff 2	S2	F	41		Personal Care Worker
		Staff 3	S3	F	50		Care Manager
		Staff 4	S4	F	-		Personal Care Worker
		Staff 5	S5	F	60		Enrolled Nurse
		Staff 6	S6	F	52		Diversional Therapist
		Staff 7	S7	F	30		Endorsed Enrolled Nurse

### Indicators of feasibility

#### Acceptability and implementation

*Interviews:* Family and staff identified a number of advantages and disadvantages of the Giraff. The main advantage cited was the ability of the Giraff to reduce social isolation and increase connection by enabling residents and families to “visit” each other. This was of particular importance for participants who lived some distance away or may not have seen each other for quite some time. Families spoke to staff about their positive experiences of using Giraff and as a result staff remarked favourably on how families viewed Giraff: “*as far as them being able to look at family members that maybe they haven’t seen for years, and actually speak to them, I mean that was just incredible*” [S5]. Being able to see the family members’ face was also noted as an important aspect of maintaining residents’ connections with family members. One family member said her mother frequently forgot who she was talking to when they spoke on the telephone and often held the mouthpiece away from her mouth or upside down, making it difficult to conduct a conversation. The family member said, “*I was actually quite amazed at how relaxed*… *her mother was, it was just like a face-to-face*.” [F5b]. As one staff member noted, “*the phone it is just a voice, Giraff is a face and a voice and it’s more real”* [S5]. Similarly, communication was enhanced for one family member who said “*face-to-face conversations … are a lot easier than on the phone so this was a happy medium between the two”* [F3].

Benefits for family members included the enjoyment they got out of the experience of connecting with their relative and the reassurance it gave them to be able to see their relative was doing well and was in clean surroundings. They also found it convenient not to have to drive for long distances for a short face-to-face visit as often as they would have done if they were not participating in the trial. One family member who was babysitting her young grandchildren (the resident’s great-grandchildren) brought them into the conversation for a brief period and another family showed the resident their pet dog. In both these instances, conversation was facilitated in a way that would not have been possible without the video element of Giraff.

Benefits expressed by staff members primarily related to the enjoyment they saw residents experiencing, “*I love my job and anything that makes my residents feel better makes me feel better*” [S5]. There were no major disadvantages identified by staff or family members, however some minor issues included a blurred image on the family member’s screen due to the low quality of Giraff’s inbuilt camera, some difficulty at times with the audio volume, and the potential for family members to witness residents’ disruptive behaviours, although the staff also noted that they would likely be aware of these already and relatives did not report negatively on this. Overall, all participants wholeheartedly agreed that Giraff was a worthwhile endeavour and a “*wonderful opportunity*” [F3] for all involved.

Both staff and family members identified a number of additional uses for Giraff. One family member who lived internationally commented that it would be worthwhile having conversations via Giraff with staff members who provide regular care for their relative. This allows the family member to get more of an understanding about how the resident is doing. Additionally, staff highlighted the possibility of conducting tele-health consultations via Giraff.

The primary concern at the beginning of the trial was for how residents would react to the new technology and whether they would be confused or frightened by it. Both staff and family members highlighted this as an important concern, however all participants were pleasantly surprised at how well residents responded. None of the residents reacted adversely. Staff also indicated that fear of technology appeared to be reduced in residents not participating in the trial through observation of other residents using Giraff.

#### Implementation and practicality

*Trial Call Log*: Descriptive results for the trial calls for each of the resident-family dyads are displayed below in Table 3. Across the trial, 34 calls were scheduled, 29 scheduled calls were attempted, and 26 calls took place. Of these 26 calls, seventeen resulted in useable recordings for analysis. Data was considered to be unusable when there were several disconnections in the call or where a video recording did not record via the external camera. The shortest call lasted for approximately four minutes, whereas the longest call lasted approximately 53 minutes. The average call duration across the trial was 23 minutes (*SD* = 13.23).

**Table 3:** Success, duration and problems associated with calls for the five resident-family dyads

	**Scheduled calls (N)**	**Attempted calls (N)**	**Successful calls**^**1**^**(N)**	**Duration of successful calls (Minutes) (M (SD))**	**Calls with useable videos (N)**	**Connection problems (N)**	**Drop-outs (N)**	**Hardware problems (N)**
Dyad 1	5	5	5	33.81 (27.53)	2	1	1	0
Dyad 2	6	4	3	10 (7.07) ^2^	0	1	2	2
Dyad 3	8	7	6	26.61 (10.71)	5	2	2	2
Dyad 4	6	6	6	12.10 (6.91)	6	2	3	1
Dyad 5	9	7	6	29.90 (6.88)	4	5	2	5

Notes: ^1^Successful calls defined as those where the resident and family spoke via Giraff. ^2^Due to no usable recording for Dyad 2, estimated durations of two successful calls were obtained from research team notes.

The number of attempted calls with technical difficulties is also displayed in Table 3. Calls with connection problems included calls where there was an Internet reception problem. Calls with dropouts included calls where the connection dropped out during the call. Calls with hardware problems included those where there were issues with the Giraff program, recording software, Giraff computer, or the family member’s computer and/or modem.

The major issue faced by the research team related to Internet connection. The University owned Giraff and the long-term care facility management were unable to provide wireless Internet access due to privacy and security concerns. Therefore this resulted in connection problems being related to the environment rather than Giraff. Throughout our trial, Giraff relied on the strength of the mobile broadband reception to connect via an external modem attached to Giraff. Unfortunately the environment did not have a strong Internet signal and this was challenged further as the geographical layout of the facility buildings was not conducive to a strong Internet signal in most areas of the facility. This issue was resolved by trial and error, moving Giraff to various locations in the facility until a location was found that worked well. The poor Internet connectivity however, limited the extent to which family members could utilise the robot’s mobility feature.

A number of hardware problems were also experienced. These included overheating of the hardware due to the research team’s inclusion of extra memory, an external camera, and modem; recording file corruptions; and program errors that required Giraff to be restarted. Family members, however, did not identify these as major problems as they understood the trial nature of the project and had expected some technical problems. In the event of problems, the research team engaged the resident in conversation while Giraff was restarted.

Availability of staff to manage Giraff calls in this situation was not feasible given the technical difficulties with the external modem connectivity. All staff members, however, believed that with adequate training, scheduling, and assistance from volunteers, implementing Giraff in a long-term care facility would be feasible in the future.

#### Efficacy

**Figure 1:**
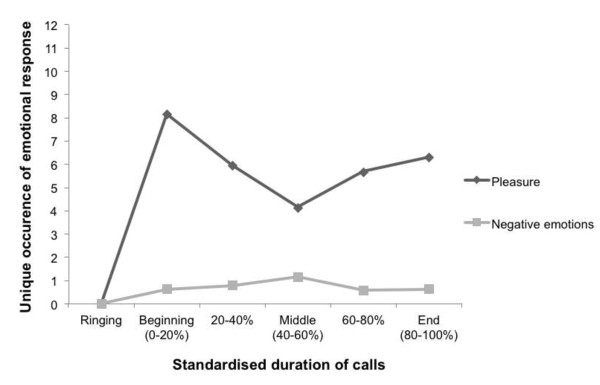
A trend diagram of positive (i.e., pleasure) and negative facial emotional responses exhibited by the residents.

Because the duration of each call session was different, it is not appropriate to report the average frequency of the emotional responses. Furthermore, residents’ emotional responses were largely dependent on the nature and content of the conversation. Therefore, a trend diagram was created to depict the frequency of positive and negative emotions across the call sessions for all residents (see Figure 1). The duration of each call was standardised into five segments (each reflecting a given 20 per cent of the call) from the start of an established call to the end. ‘Positive emotions’ reflect the occurrence of pleasure, while ‘negative emotions’ reflect the occurrence of anger, anxiety or fear, and sadness. As depicted in the trend diagram, the residents showed a general state of positive emotions and minimal negative emotions during the call.

Residents tended to exhibit the greatest positive emotions at the beginning of the call when they first saw their family member on Giraff’s screen. Positive emotions tended to taper off in the middle of the call, but residents were engaged in the conversation, and showed instances of pleasure consistent with the nature and content of the conversation. A slight increase in positive emotions was also observed near the end of the call when family members made arrangements for the next call. Most residents waved good-bye to their family member and one resident (Dyad 3) blew kisses to his daughter at the end of each call. Overall, the residents appeared to enjoy the conversation and the interaction with their family members.

Residents were also coded to have a high level of engagement (i.e., visually alert and verbally engaged) during the calls (see Table 4). Residents showed consistent average engagement for 93 per cent (*SD* = 15%) of the call duration. Resident 3 (R3), however, showed low levels of engagement in Call 2 and Call 3. This appeared to be related to his advanced state of dementia, his spouse being present with him at the facility during the calls, and the spouse leading the conversation with the daughter (who was calling via Giraff). In this case, the conversation was taking place between the daughter and spouse, rather than engaging Resident 3. With the exception of these calls, all dyads were noted to have actively participated, encouraged, and maintained conversations with each other and used Giraff’s video capability to enhance the conversation with references to the environment and other visual stimuli. For example, in one of the call sessions, a family member (Dyad 3) virtually drove Giraff around to examine the resident’s living environment. The drive-around allowed the family member to experience and talk about the room fittings. This demonstration also encouraged the resident (R3) to laugh and question the family member about the mobility of Giraff. On average, dyads used the opportunity for visual cues 7.26 times (*SD* = 4.59) in each call session to engage with the family member.

**Table 4:** Engagement (proportion of call) and visual cues (number of unique instances) exhibited by residents

		**Call 1**	**Call 2**	**Call 3**	**Call 4**	**Call 5**	**Call 6**
Dyad 1							
	Engagement (%)	98	100	-	-	-	-
	Visual Cues (n)	6	9	-	-	-	-
Dyad 3							
	Engagement (%)	100	41	73	92	100	-
	Visual Cues (n)	11	17.5	11.5	2	7	-
Dyad 4							
	Engagement (%)	100	100	100	100	90	100
	Visual Cues (n)	11	9	4	3	0	0
Dyad 5							
	Engagement (%)	92	97	99	99	-	-
	Visual Cues (n)	10	9	8.5	5	-	-

Note: - indicates that there was no usable recording for the analysis.

## Discussion

The aim of this study was to determine the feasibility of using Giraff to connect and engage people with dementia living in long-term care with their family. Using an adaptation of the Bowen Feasibility Framework [17], findings from this study showed that Giraff was acceptable; was feasible to be implemented within the long-term care facility, although there were several technical issues that impacted on its implementation; and showed trends in efficacy. For families and the person with dementia, being able to see each other via Giraff’s large video screen appeared to enhance communication by increasing the naturalness of, and resident engagement in, the conversation. During the calls the researchers noted that engagement in the conversation was seen to be enhanced when residents and family members were able to comment on what they could see or show each other via the robot’s video screen. Such items provided reminiscence opportunities and therefore conversation points for the resident and family. The ability to move Giraff around the resident’s room as well as the facility enabled families to observe their relative’s environment and if desired, to follow the resident around the facility so they could point out objects that they found to be of interest.

The impact of Giraff on residents and their families was very positive. At the beginning of the trial, family members were very excited about the opportunity it afforded to see their relatives. Family members also reported feeling reassured knowing their relative could participate in the calls sitting in the comfort of their own room or in the lounge, rather than at a computer desk that might occur with Skype or a similar program.

At a cost of around $10,000 (US) per robot there is a need to also consider the cost effectiveness of Giraff. Prior to the trial the research team were asked why we were testing an expensive robot to engage people with dementia with family rather than using iPad and Skype software to complete the connection. An advantage that Giraff has over Skype or an iPad is that it is the family who has control in terms of connecting to their relative, virtually driving Giraff and positioning the video camera so that both the family and resident can see each other as well as observe items of discussion within the environment. This in effect takes the stress off the person with dementia in needing to recall or learn a new skill such as using an iPad or Sykpe and ensures that the video camera can be positioned appropriately to enhance communication. This was supported by two of the family members who discussed with the team their previous experience of trying to use an iPad to talk with their family member. They acknowledged that Giraff was much easier than using an iPad as their experience had demonstrated the inability of the person with dementia to be able to hold or adjust the iPad so that the iPad camera could identify the family member. An additional benefit, highlighted as an issue for one resident, was that Giraff could not be ‘misplaced’ by the resident after a call, as was often the case with the telephone (and possibly an iPad if being used for Skype calls). Future research will help to unravel such problems in a future study that will compare iPad and different types of telepresence robots.

During the research and as a means to maintain privacy, Giraff was only used at times prearranged with the resident, family and staff. Outside of these times Giraff was turned off and was only accessible when switched on by the team and by the family member given access to the software. The resident also had the opportunity to terminate the call at anytime by using a large red stop button positioned on Giraff. Privacy needs to be carefully considered when setting up telepresence robots within the care environment.

Prior to the research, the team and others were unsure of how a person with dementia would react to the large blue coloured robot. It was interesting to note that participants did not display concern about Giraff, and other residents were inquisitive about Giraff, often spending time during the ‘test runs’ observing Giraff and the family conversations.

The research team reported the technical challenges experienced to the developer of Giraff. The recently released next generation Giraff has been further developed to overcome these technical challenges. Connectivity however, will continue to be a challenge in situations where broadband access is limited. The long-term care site chosen to conduct this trial was in an area described as having adequate internet reception and Internet access speed, however, the site was challenged by connectivity access, even to the point where staff were unable to use mobile telephones in many parts of the building. This access problem could have been reduced if the long-term care setting had allowed access to the facility Wi-Fi rather than the team needing to rely on mobile broadband access. In the future researchers may need to resolve the facility’s privacy and security concerns to enable access to the long-term care facility’s Internet access, and long-term care settings may need to upgrade their Internet access if they decide to purchase a telepresence robot.

Although we explored the use of the telepresence robot with family and the person with dementia, the project raises a number of other potential opportunities where Giraff could assist. In particular, and as indicated by family, Giraff could be used to inform family of the resident’s condition, to put a face to the voice of a staff member, and for the family member to feel more involved and connected with the facility. Other uses could include inter-facility communication via Giraff to enable residents with friends in other facilities to communicate with each other and to maintain social connections outside of the facility. However, future studies must include a cost analysis of Giraff to enable long-term care facilities the opportunity to weigh up the benefits of Giraff with the human and physical costs both for the initial purchase and maintenance.

The mixed method approach and feasibility framework allowed opportunities to collect various data formats and to review these using different methods. People with dementia, in particular those with Alzheimer’s disease, usually have difficulty in decoding emotions cognitively, such as recognising and comprehending emotions displayed by others and a diminished ability to express their feelings verbally [24]. This can result in people with dementia expressing emotions through facial expressions and gestures. Therefore, recognition of facial expression is considered to be one of the prominent non-verbal means of understanding for example an expression of distress or pain [25]. Although facial expression has received a lot of interest in research with people who are not cognitively impaired, there has been limited research exploring the display of emotion (encoding) in persons with dementia. This current research is therefore important in demonstrating the significance of video observation in understanding the impact of an intervention, in this case, a telepresence robot.

The small number of participants limits this study however the focus of the study was on feasibility rather than generalisability. Therefore a small purposive sample was appropriate to examine feasibility.

## Conclusions

This study used a mixed method approach and a feasibility framework to examine the feasibility of an innovative telepresence robot to enhance engagement between family and a person with dementia living in long-term care. Participants perceived Giraff as a positive and therapeutic option to engage people with dementia with their family member. In spite of the technical difficulties families and staff saw the advantages of Giraff and viewed the positive reactions of the residents to their connection with family via Giraff. The new improved second generation Giraff has been developed to overcome the technological challenges experienced by the researchers. Therefore, with this in mind the findings support the need for a larger trial, for a longer time period using the second generation Giraff and the inclusion of a cost analysis.

## Abbreviations

TRIC: Telepresence Robot for Interpersonal Communication; ExCITE: Enabling social interaction through embodiment research project; MCI: Mild cognitive impairment; OERS: Observed emotion rating scale; R1 to R5: Resident Pseudonyms; F1 to F6: Family Pseudonyms; S1 to S7: Staff Pseudonyms.

## Competing interests

The authors declare they have no competing interests.

## Authors’ contributions

WM conceived of the study and CJ, MC, SOD and BS were involved in the design. SD and BS undertook acquisition of data and CJ, BS, SD and WM undertook analysis and interpretation of data. WM drafted this version of the manuscript and all authors were involved in revision of the manuscript. All authors read and gave final approval for this version of the manuscript to be published and agree to be accountable for all aspects of the work.

## Pre-publication history

The pre-publication history for this paper can be accessed here:

http://www.biomedcentral.com/1471-2318/14/7/prepub
